# Modeling current and future global distribution of *Chrysomya bezziana* under changing climate

**DOI:** 10.1038/s41598-020-61962-8

**Published:** 2020-03-18

**Authors:** Eslam M. Hosni, Mohamed G. Nasser, Sara A. Al-Ashaal, Magda H. Rady, Mohamed A. Kenawy

**Affiliations:** Department of Entomology, Faculty of Science, Ain Shams University, Abbassia, Cairo, 11566 Egypt

**Keywords:** Entomology, Biogeography

## Abstract

In the last few years, significant changes in climate have had a disparate effect on biodiversity. The influences of these changes are random and unpredictable. The resurgence of insect pests, especially of medical and veterinary importance, often corresponds with climate changes. The Old World screwworm, *Chrysomya bezziana*, is one of the most important myiasis-causing flies that parasitize warm-blooded animals in the Eastern Hemisphere. We used a spatial distribution modeling approach to estimate the consequences of climatic changes on the potential geographic distribution of this insect throughout the world currently and in the future. A Maxent model used occurrence data from 104 localities and 19 climatic factors to predict the suitable habitat regions throughout the world. Two representative concentration pathways 2.6 and 8.5, were used to forecast the future distribution of *C. bezziana* in 2050 and 2070. The Maxent model for *C. bezziana* provided a satisfactory result, with a high value of the Area Under Curve equal to 0.855 (±0.001). Furthermore, the True Skilled Statistics value is equal to 0.67. These values indicate the significant influence on the model of the ecology of this fly species. Jackknife test indicated that temperature variables play a significant role in *C. bezziana* dynamics. The resultant models indicated the areas at risk of invasion by potential serious medical/veterinary issues, especially in countries with a large livestock production.

## Introduction

Throughout the long history of our planet, the climate has changed dramatically, but in the last few decades global warming has become more tangible even for the layman^1,2^. Greenhouse gases emitted as a result of anthropogenic action are the main factors driving global warming^3^. The Intergovernmental Panel on Climate Change (IPCC) has predicted an increase of about a 1.8–4 °C in global temperature by the end of the 21^st^ century as a result of high CO_2_ levels^4^. This form a challenge for many ecosystems throughout the world, threatening ecological processes and impacting on biodiversity, including insects^5^. Conversely, climate change is one of the most important factors associated with the resurgence of insect pests. Many medically important pests such as mosquitoes (Culicidae) will invade new regions because of changes in global temperature^6^. Flies that cause myiasis will also be able to invade new regions and impact the livestock economy in different parts of the world.

Myiasis is a type of parasitism in which the living tissues of a vertebrate host are infested by dipterous larvae^7^. This phenomenon is widespread throughout the world, especially the tropical regions. It usually occurs in both domestic and wild animals and, under certain conditions, in humans^8,9^.

The Old World screwworm fly (OWSF) *Chrysomya bezziana* (Villeneuve) is an obligate parasite causing myiasis in animals and humans in the Eastern Hemisphere^7,9,10^. Females deposit their eggs in wounds or near the natural body orifices of the targeted host. Then the maggots burrow and feed on the living tissue, causing severe injury and even death in untreated cases^11^. As a result of this feeding habit, *C. bezziana* is considered to be a primary pest of great medical importance and the cause of economic losses among domesticated animals^9,12^. In the Eastern Hemisphere, particularly in Iraq in the 1990s, the FAO estimated the economic loss to the livestock industry as a result of the OWSF invasion to be US$ 8,555,000^13^. Other research studies have estimated the annual losses in the livestock industries in Australia to be equal to A$500 million due to the possible incursion of the OWSF^14^.

To date, OWSF prefers tropical and subtropical climates. It is widespread throughout tropical and Sub-Saharan Africa, the Middle East region, the Indian subcontinent, from Southeast Asia to China, and the Philippines to Papua New Guinea^15,16^. Recently, OWSF has been expanding out of its typical range through the commercial movement of livestock and possibly also due to climate change^17^. The distribution of OWSF in the Eastern Hemisphere and of the ecologically convergent New World screwworm fly (NWSF) *Cochliomyia hominivorax* (Coquerel) in the Western Hemisphere is usually limited by temperatures^10^.

On several occasions, NWSF has been transported to the Eastern Hemisphere^10^. For example, a veterinary record about a case of otitis in a dog was recorded in 1982^18^. In 1988, an accidental introduction of NWSF occurred from an endemic area of South America to Libya^10,19^. Three years later, a wide range of infested animals were detected in about 25,000 km^2^ around Tripoli, Libya^20^. The FAO released a large-scale control program for the eradication of NWSF via the sterile insect technique, with a cost of US$750 million^17^. Intensive surveillance and prevention programs were set up by official Egyptian veterinary official institutions to prevent the accidental introduction of NWSF from the western border with Libya^19^. Although no cases of myiasis caused by OWSF have been recorded in the Western Hemisphere, about four species of *Chrysomya* have been established in the New World^21^.

The negative impact of environmental challenges is becoming the main obstacle for the sustainable development of human health and the livestock industry. Climate change forms one of these challenges, and predicting the relationship between species diversity and climate changes has become an active research issue^22,23^. Also, predictions of the scientific community and decision-makers to the future risks of pest introductions, allow them to create a driven strategy to face climate change impacts on biodiversity and medical sectors^24^.

Spatial- and temporal-based models could establish monitoring programs that act as early warning signs during the process of climate change^25^. Species distribution modeling (SDM) is a method that predicts and describes the precise niche of each species^26,27^. This can be achieved by using the presence-only data and assumed environmental variables^28^. CLIMEX, GARP, HABITAT, and MaxEnt are popular tools used to estimate the current and future distribution of targeted species under different climate change scenarios^29–31^. Predictive models of economically important livestock and human health pests are considered to be one of the most urgent direct goals of such modeling techniques^3,32^. The objective of the present research is to predict the potential spatial distribution of OWSF throughout the world by using the geographic information system and bioclimatic variables for its current and future status.

## Methods

### Occurrence records

Almost all available records of OWSF were collected from the literature either from adult flies or from medical and veterinary larval cases^23,33–35^. The records of OWSF in the digital database (www.cabi.org) were also considered^13^. The occurrence data from 104 localities were converted into comma-delimited (CSV) formats and used to assess habitat suitability for OWSF throughout the world (see Supplementary dataset).

### Climatic data

Bioclimatic data were obtained from (www.worldclim.org) with a spatial resolution of approximately 5 km^2^. A total of 19 climate variables, originally derived from monthly temperature and rainfall values collected from weather stations in 1950–2000, were used to depict the current global climate (Table 1 & see Supplementary information S1). These layers were converted to ASCII format using ArcGIS v 10.3 and used to evaluate the most important variables which contribute biologically to the current model of *C. bezziana* habitat suitability. Bioclimatic variables 8–9 and 18–19 were eliminated from the analysis, due to spatial artifacts in those variables^36^: these layers showed odd discontinuous spatial anomalies between neighboring pixels which lead to lack of resolutions in resulted layers^37^. For future data, we used parallel datasets for global climate model (GCM) from two representative concentration pathways (RCPs) 2.6 and 8.5 to account for the future distribution of OWSF based on carbon dioxide emission for 2050 (average of predictions for 2041–2060) and 2070 (average of predictions for 2061–2080)^38–40^ (see Supplementary information S2). These future data layers were also converted to ASCII format via ArcGIS v 10.3. Then the climatic data of the selected variables was projected on to the years 2050 and 2070 from the global climate model of the Meteorological Research Institute (MRI-CGCM3), to assess the effects of climate change on the OWSF habitat suitability in the future. These data are among the recent GCM climate projections that are used in the Fifth Assessment IPCC Report.

**Table 1:** Bioclimatic variables used in Maxent to predict the preliminary model of the current distribution of *Chrysomya bezziana*.

Variable	Description
Bio 1	Annual mean temperature
Bio2	Mean Diurnal Range (Mean of monthly max temp – min temp)
Bio 3	Isothermality (bio2 / bio7) (*100)
Bio 4	Temperature Seasonality (standard deviation *100)
Bio 5	Max Temperature of Warmest Month
Bio 6	Min Temperature of Coldest Month
Bio 7	Temperature Annual Range
Bio 8	Mean Temperature of Wettest Quarter
Bio 9	Mean Temperature of Driest Quarter
Bio 10	Mean Temperature of Warmest Quarter
Bio 11	Mean Temperature of Coldest Quarter
Bio 12	Annual Precipitation
Bio 13	Precipitation of Wettest Month
Bio 14	Precipitation of Driest Month
Bio 15	Precipitation Seasonality (Coefficient of Variation)
Bio 16	Precipitation of Wettest Quarter
Bio 17	Precipitation of Driest Quarter
Bio 18	Precipitation of Warmest Quarter
Bio 19	Precipitation of Coldest Quarter

### Habitat suitability modeling

The maximum entropy algorithm implemented in Maxent v3.3.3e was used to estimate the habitat suitability and ecological niche of *C. bezziana*^41^. This algorithm yields an excellent predictive models that rely on presence data only^26,41^. Also, It is effective in modeling studies with small sample sizes and is able to remove the duplicate records in the same cell^26,42^. Additionally, the response curve for each bioclimatic variable was used to estimate the relationship between the habitat suitability for a species that varies from 0 (lowest suitability) to 1 (highest suitability) and bioclimatic variables^43^. In our models, 75% of the occurrence records were used for training whereas 25% of the records used for testing the model^44^. The maximum number of background points and the number of iterations was 10,000 and 1000 respectively. Furthermore, the process was repeated in a 10-fold cross-validation, which improved the model performance^31,44,45^.

**Figure 1:**
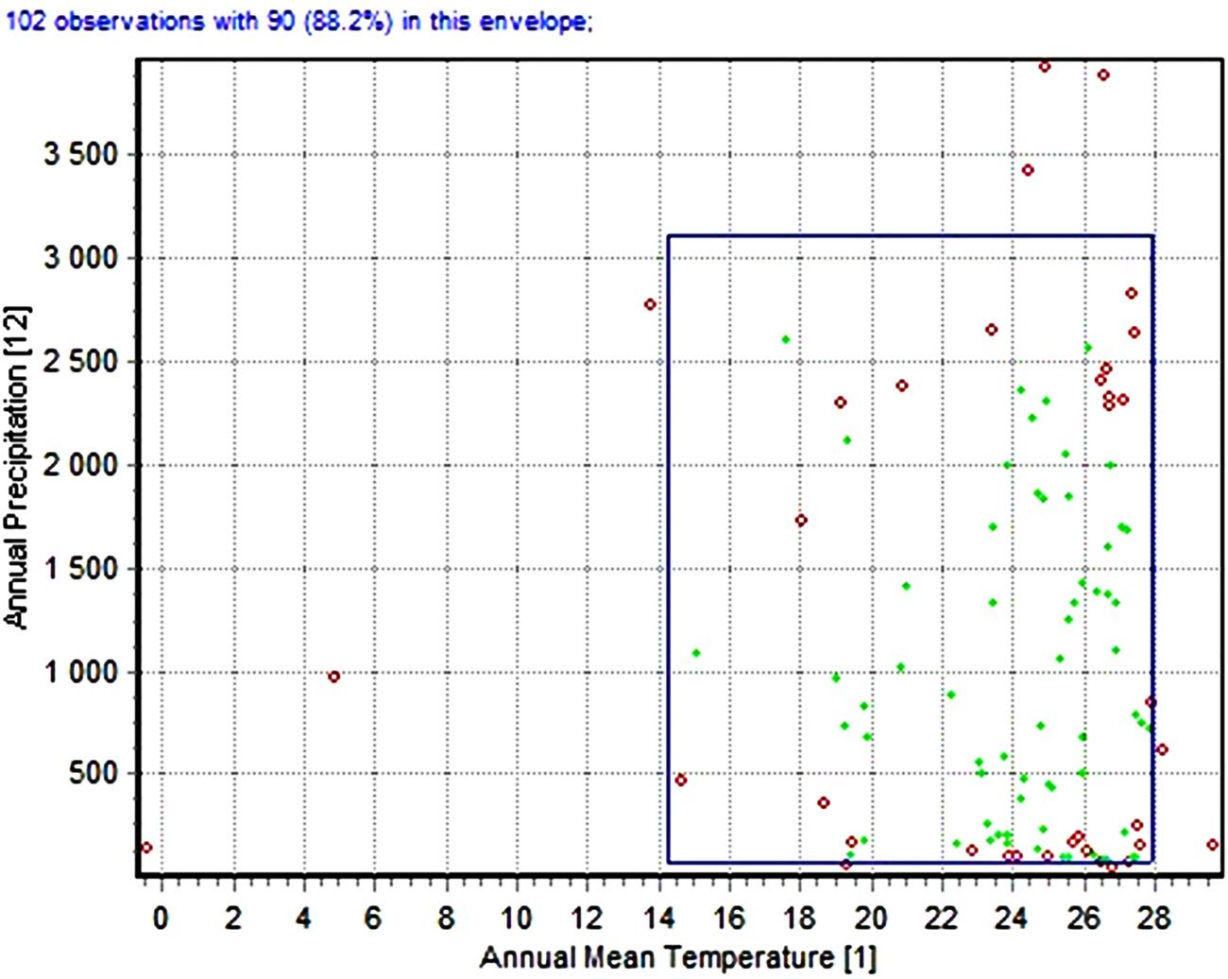
Environmental envelope model of recorded points of *Chrysomya bezziana*, the envelope showing the wide range of Annual precipitation (Bio 12) against an effective small range of Annual mean temperature (Bio 1).

A set of five biologically significant bioclimatic variables was chosen to produce the final models based on several statistical analysis: (Bio 1) Annual mean temperature, (Bio 3) Isothermality, (Bio 6) Min temperature of the coldest month, (Bio 10) Mean temperature of the warmest quarter and (Bio 11) Mean temperature of the coldest quarter. First, the Jackknife function of MaxEnt was used to estimate the most important set of variables from the 19 bioclimatic factors by discarding the variables contributed less than 70% (see Supplementary Fig. S3). Species Distribution Modeling (SDM) Tools in ArcGIS 10.3 (Universal tool; Explore climate data; Remove highly correlated variable) were then used to remove variables with high correlations^46^. As the five most important variables were related to temperature, the environmental envelope test evaluated the impact of precipitation variables on the distribution of *C. bezziana* by using DIVA-GIS 7.5 software (Fig. 1). The generated graph indicated that the precipitation variables show less or no contribution to *C. bezziana* distribution as 88.2% of all records used in the model occur on the envelope which has the expanded range of Annual precipitation (Bio 12) in contrast to the narrow range of annual mean temperature (Bio 1). Such analysis indicates the null effect of all precipitation variables on the final models.

### Model evaluation

The area under curve (AUC) of the receiver operating characteristics (ROC) was used to estimate the model performance, and its value varies from 0 (random discrimination) to 1 (perfect discrimination). Higher AUC values suggested significant influences of the model with the ecology of OWSF. Values less than 0.5 indicated poor-fitting models while AUC values more than 0.75 indicated high-fitting of the models^47,48^. In addition, the True Skilled Statistics (TSS), a common statistical method, was used to evaluate the accuracy of predicted models. TSS values range from -1 to 1 and values that are close to negative or zero imply that the distribution is not much better than random, while values close to 1 indicate an intimate relationship between the model prediction and distribution^49^.

### Ethical approval

This article does not contain any studies dealing directly with animals, and all applicable international, national and /or institutional ethical guidelines were taken in consideration during preparation of this manuscript.

## Results

The occurrence data of OWSF were taken from 104 localities throughout the World. Generally, OWSF occurs in tropical and subtropical climates. Most of the occurrence records were geographically distributed across Africa and Asia.

### Modeling performance

The Maxent model for OWSF provided a high value of the Area Under Curve (AUC) equal to 0.855 (±0.001) (see Supplementary Fig. S4). In continuous species distribution modeling, the values of AUC tended to be high rather than discontinuous. Also, model performance tended to be excellent due to the high value of the true skilled statistics (TSS) equals 0.67. Normally, TSS values of ≥0.5 are acceptable.

### Contribution of bioclimatic variables

**Figure 2:**
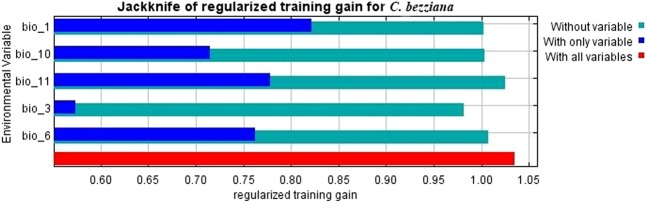
Jackknife test for *Chrysomya bezziana* showing the most effective environmental variables.

**Figure 3:**
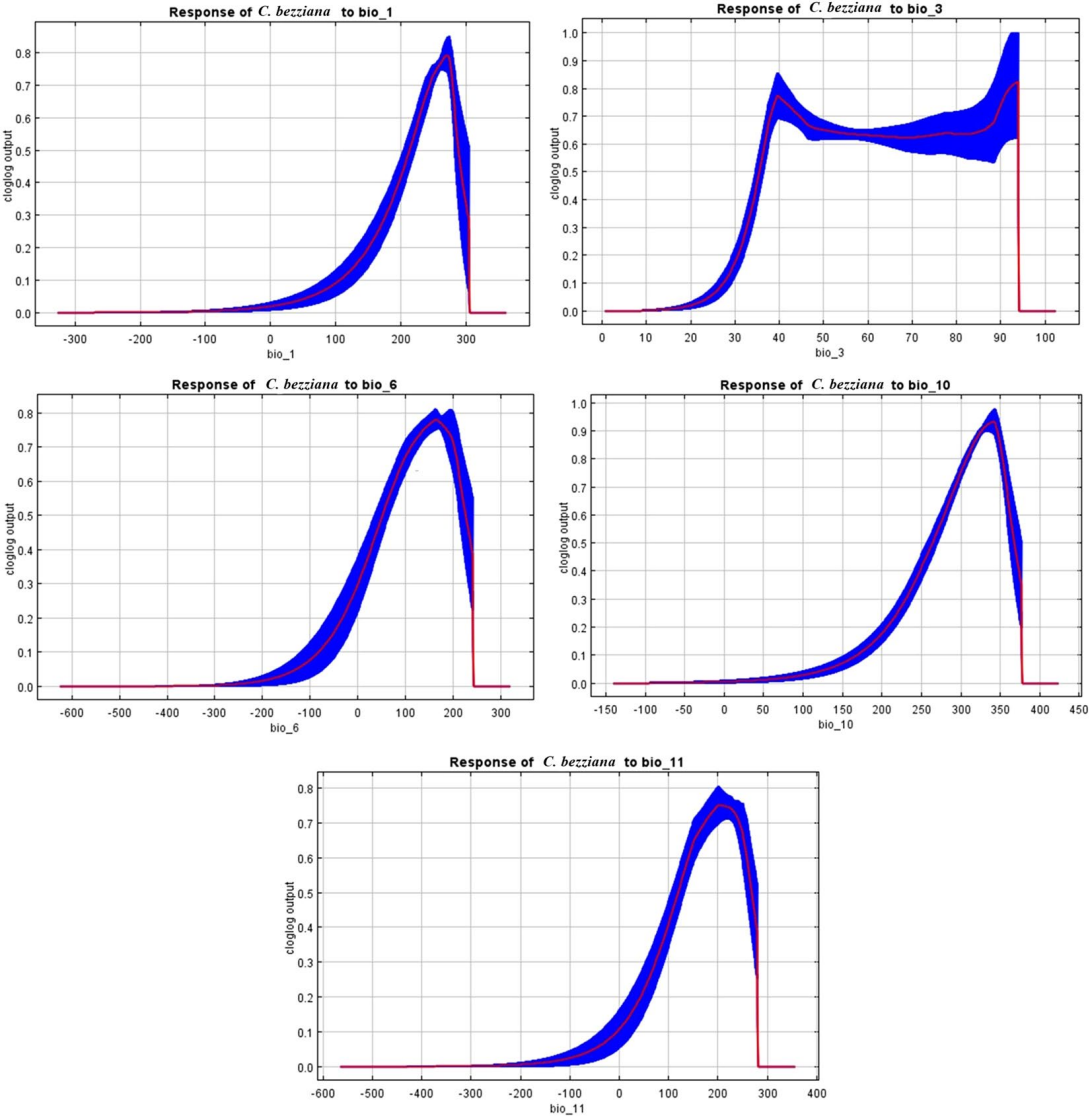
Response curves of the most relevant environmental factors affecting the distribution of *Chrysomya bezziana;* the shown values are average of ten replicate runs.

The jackknife test illustrated the contribution percent of bioclimatic variables of the predictive distribution model (Fig. 2 and Table 2). Annual mean temperature (Bio 1) made the largest contribution to OWSF distribution with 48.4%, followed by Mean Temperature of Coldest Quarter (Bio 11), Mean Temperature of Warmest Quarter (Bio 10), with 30.1% and 9.9 respectively. The remaining variables, Min Temperature of Coldest Month (Bio 6) and Isothermality (Bio 3), had only a minor effect with 6.1% and 5.5%, respectively. Response curves of the most contributory environmental variables (Fig. 3) suggested that the favorable annual mean temperature of OWSF ranged from 28 °C to 30 °C. Notably, Bio 11 and Bio 10 critically affected OWSF distribution.

**Table 2:** Relative percentages of bioclimatic variables used in Maxent to model the current and future habitat suitability of *Chrysomya bezziana*.

Bioclimatic variables	Description	Contribution percentages
Bio 1	Annual mean temperature	48.4%
Bio 11	Mean Temperature of Coldest Quarter	30.1%
Bio 10	Mean Temperature of Warmest Quarter	9.9%
Bio 6	Min Temperature of Coldest Month	6.1%
Bio 3	Isothermality (bio2 / bio7) (*100)	5.5%

### The Predicted current potential distribution

**Figure 4:**
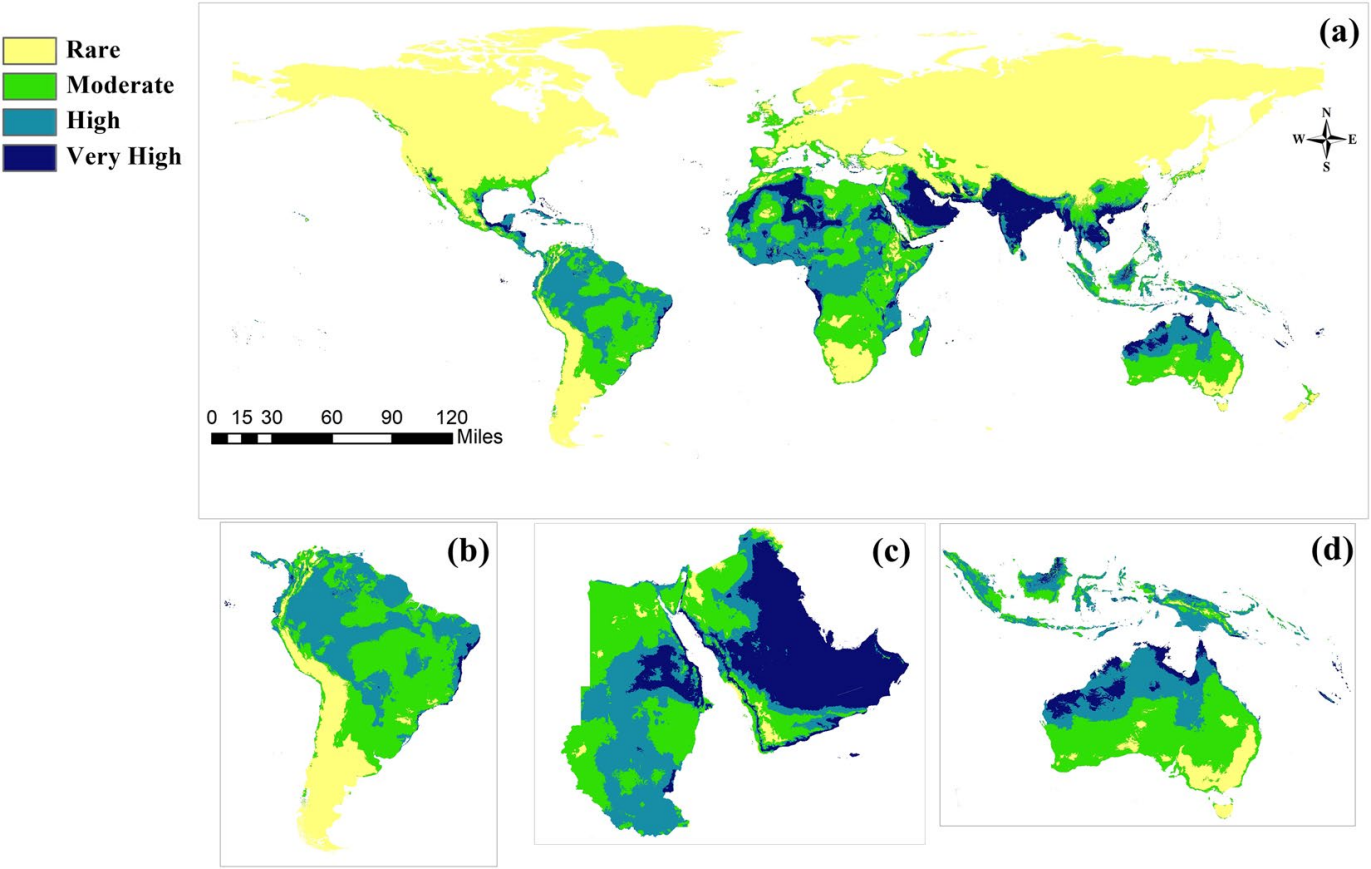
Current potential distribution of *Chrysomya bezziana* with three zonation areas in South America, the Middle East region and Australia.

The predictive model generally agreed with the occurrence records in regions that already have OWSF infestations. In Africa, our predicted model is compatible with the real distribution of the fly from the Horn of African to Sierra Leone. It also shows some other areas with a high existing probability such as Niger, Algeria, and Mali (Fig. 4a).

In Asia, the potential distribution of OWSF occupies most of the currently-infested areas. The Indian subcontinent, Southeast Asia (Indonesia, Malay Peninsula, Malaysia and Philippine Islands) and Papua New Guinea (PNG) showed a high to very high habitat suitability, respectively (Fig. 4a). On the other hand, central and northern Asia indicated a high level of distributional limitations.

In Europe, geographical barriers have prevented the movement of OWSF. However, the southern areas of Europe along the Mediterranean Sea, especially the Greek islands, Malta, and southern Italy, showed high habitat suitability (Fig. 4a).

In the Americas, the results indicate areas that were predicted as suitable but are not currently occupied by OWSF: coastal Florida, Cuba, and the Bahamas displayed high habitat suitability (Fig. 4a). The model also showed a high suitability in the uplands of the Amazon River Basin, coastal Brazil along the Atlantic Ocean and the Galapagos Islands of Ecuador (Fig. 4b).

In the Middle East, OWSF is endemic in the Kingdom of Saudi Arabia, the United Arab Emirates, the Sultanate of Oman, Iraq, Iran, and incidentally in Bahrain, Kuwait, and Yemen. Our model confirmed this distribution and indicated other areas with a very high habitat suitability in the Hejaz west of Saudi Arabia, the mountains of Sarawat and the coastal region of Yemen along the Indian Oceans. The OWSF is not currently established in Egypt. However, the northern strip along the Mediterranean Sea had a high habitat suitability. Also, the coastal regions of the Nile Delta and the coastal strip of the Red Sea in Sinai exhibited a very high habitat suitability (Fig. 4c).

Australia is the only continent with a tropical zone without a true invasion of OWSF. The current distribution model (Fig. 4d) indicated a very high habitat suitability in the northern strip, especially at Port Darwin. A high habitat suitability is also evident in the Northern Territory and parts of Queensland. The Fiji island, New Caledonia and the Solomon Islands show a very high habitat suitability for the fly without any true records until now.

### The Predicted future potential distribution for 2050 and 2070

**Figure 5:**
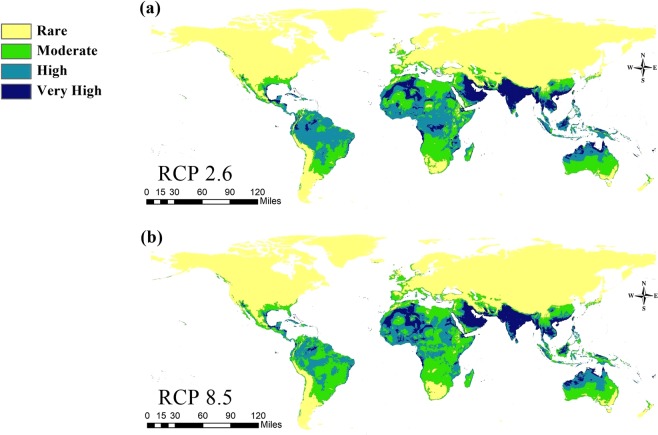
Predicted future distribution of *Chrysomya bezziana* under two representative concentration pathways (RCPs 2.6, 8.5) of climate conditions in 2050.

**Figure 6:**
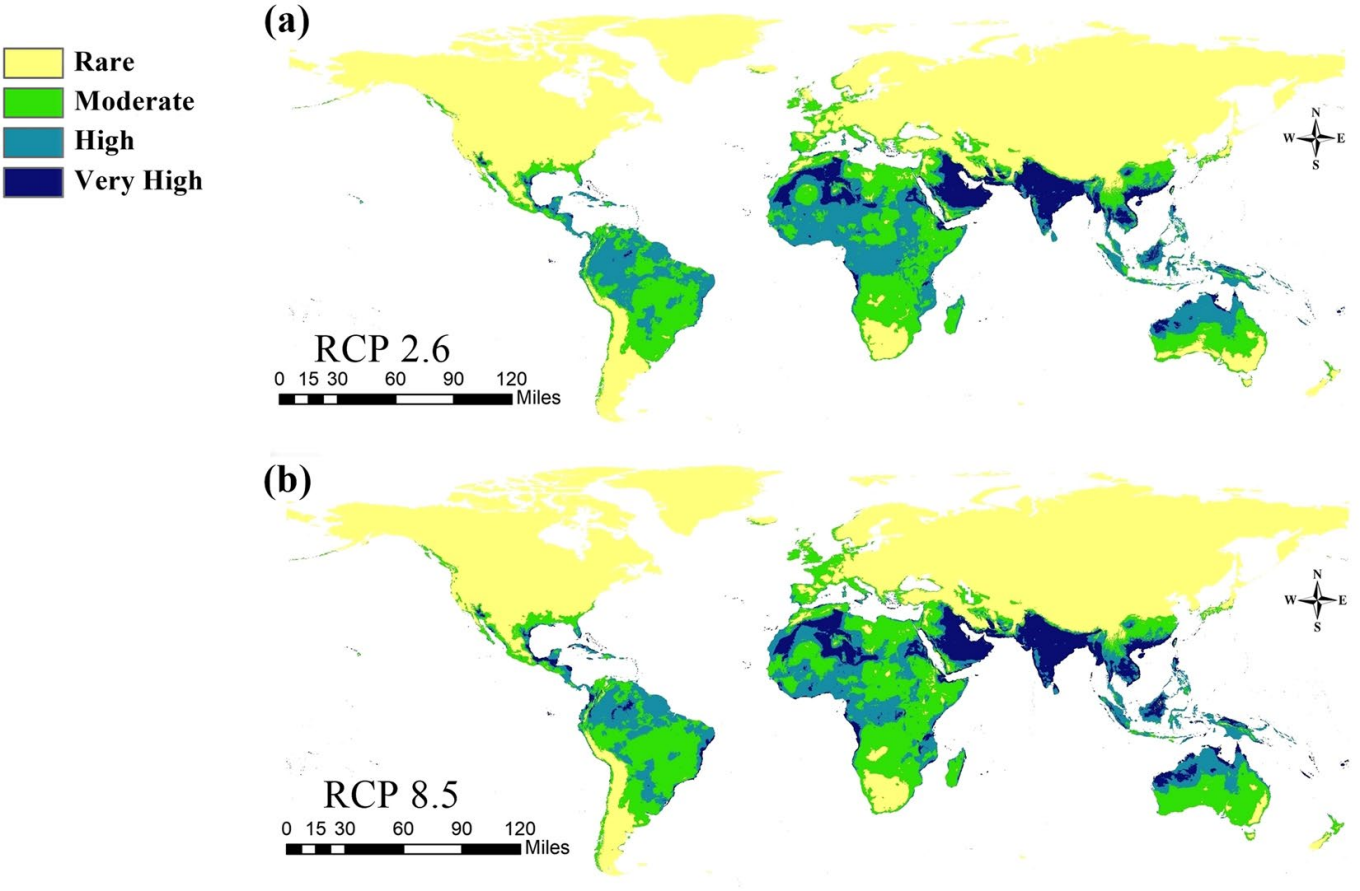
Predicted future distribution of *Chrysomya bezziana* under two representative concentration pathways (RCPs 2.6, 8.5) of climate conditions in 2070.

The overall distribution patterns throughout the Old World between the present-day and future models showed reasonable similarities except in some regions. Furthermore, the future predictions showed some differences between RCPs in 2050 and 2070 (Figs. 5, 6).

For the time period 2050, except for the African Sahara which showed a low suitability, the future model in RCPs 2.6 and 8.5 confirmed a high habitat suitability in the Horn of African, Sub-Saharan Africa and West Africa. In Asia, endemic regions (Saudi Arabia, United Arab Emirates, Sultanate of Oman, Iraq, Iran, Bahrain, Kuwait, and Yemen) and the Indian subcontinent, Southeast Asia and PNG showed a very high suitability (Fig. 5a,b). For RCPs 2.6 and 8.5 during 2070, the predictive model illustrated a dramatic change in OWSF distribution and habitat suitability in the African continent. Nevertheless, the endemic regions of Asia, the Indian subcontinent, and South east Asia still show a very high habitat suitability (Fig. 6a,b). Interactive calibrated maps show the gain and loss of the overall habitat suitability differences among current and future models are provided here as supplementary materials (see Supplementary Figs. S5–S9).

## Discussion

Throughout the world, myiasis is considered to be a neglected health and veterinary concern^50^. The Old World screwworm fly is one of the most important myiases-producing agents^7^. It poses a great threat to the livestock industry in many parts of the world^10,15^. The present study provides updated and detailed maps about the current and future global distribution of OWSF in the light of climate changes. Our generated maps are of public health and veterinary importance as they estimate the potential distribution of OWSF in the Eastern Hemisphere and predict susceptible regions of invasion outside the actual currently-known distribution of this fly. These model maps also illustrate the influence of climate changes on OWSF distribution. Furthermore, these findings can assist concerned international organizations and decision-makers to assess surveillance and control programs for OWSF.

Only one previous study compared the global distribution of OWSF with the predicted distribution using CLIMEX in relation to climatic factors^15^. Another powerful and updated predictive tool is Maxent. Maxent models are characterized by their robust predictive powers for the potential distribution of species^51^. Also, its statistical algorithm is accurate even with small occurrence records. Our Maxent model showed that the predicted habitat suitability agreed strongly with the actual occurrence of OWSF records with a high AUC value, which suggests a close relationship between the model and the ecology of the species. Moreover, the TSS value of 0.67 illustrated a perfect agreement between the model predictions and fly’s distribution.

Previous studies emphasized that temperature is the most significant variable that affects OWSF distribution^14,15^. Our model agrees with this finding, and the ambient temperature variables were the most effective bioclimatic variables of jackknife test that affected OWSF distribution. These variables are Annual mean temperature (Bio 1), with the largest contribution to OWSF distribution (48.4%), followed by Mean Temperature of Coldest Quarter (Bio 11) and Mean Temperature of Warmest Quarter (Bio 10) with 30.1% and 9.9%, respectively. The remaining variable was Minimum Temperature of Coldest Month (Bio 6) with 6.4%. The cumulative contribution of these variables equals 94.8% (Table 2, Fig. 3). All response curves of the five temperature bioclimatic variables show an increase in habitat suitability of fly up to a certain point at which a further increase in temperature could eliminate the fly from the ecosystem.

The current model for the potential distribution of the OWSF is closely associated with present-day distributions except for minor changes. Old World screwworm flies naturally occupy tropical and subtropical areas of Asia and Africa. Even a sudden increase of 3 °C in annual mean temperature in different eco-climatic zones of the Old World is considered tolerable for OWSF (Fig. 3). Only in the middle and northern Asia were there considerable distribution limitations of OWSF due to low temperatures in the coldest quarter. Our model highlighted areas with large livestock production, such as Brazil and the northern regions of Australia, as being of high and very high habitat suitability even though they are still free from this fly.

With the influence of climatic change, the potential distribution and population dynamics of OWSF were greatly affected by rises in temperature. The future GCM models of 2050 and 2070 with RCPs 2.6 and 8.5 illustrate a high risk in areas with a large livestock production. This also agreed with the previous CLIMEX model of the potential geographic distribution of OWSF^15^. Both models showed a high suitability in the Western Hemisphere, especially in Brazil and an undoubted high risk in Australia associated with the movement of animals within the countries of Southeast Asia and PNG.

Australia forms a special case for OWSF invasion risk. The country is close to the fly risk areas in the oriental region. The current and future models show a high and a very high suitability of northern Australia to OWSF. In the real world, Australia has already faced some cases of OWSF detection on quarantines on northern harbors due to livestock trades including a collection of adult male and female flies^52^. So, the resultant models act as a warning guard for Australian quarantine authority to avoid the possible invasion of OWSF to their northern territory^14^. A similar status of incursion has occurred in Libya with NWSF^20,53^, the FAO estimated the cost of eradication-via a sterile insect technique- to reach US$750 million^17^.

In Africa, the effect of climate change is unequivocal (Figs. 5, 6). The future models showed that the increase in temperature affected the distribution of OWSF negatively because of a low habitat suitability, especially in Central and Southern Africa. Moreover, the occurrence of OWSF will be restricted to the region of West Africa and the Horn of African (Figs. 5, 6).

Across Asia, all future models confirmed that the Indian subcontinent, Southeast Asia, the southern parts of China and PNG have a high suitability for OWSF (Figs. 5, 6). In other areas in the Middle East, the endemicity of flies increased because of a high suitability. This agreed with the FAO report about the geospatial demarcation of OWSF in the Middle East^51^. This report confirmed the influence of temperature on the potential distribution of OWSF in the Middle East.

The future models for 2050 and 2070 also indicated the risk of future invasions into southern part of North America and South America. In the same way, islands with a fragile ecosystem, an economy based on livestock trades or low population densities, such as the Bahamas, Fiji island, Galapagos, New Caledonia, Socotra and the Solomon Islands, have a high habitat suitability for OWSF invasion (Figs. 5, 6). Effective planning by quarantine authorities, especially in such sensitive ecosystems, is very important to avoid economic damage.

Our study gives an important insight into the current and future status of *C. bezziana* throughout the world. The distribution models generated in this study, based only on climatological variables, assess the effect of climate change on the current and future distribution of OWSF. Several publications have used only the climatic variables for this purpose^54–59^. Incorporating other environmental variables such as human population, land cover, vegetation index, host animal distribution, etc, could improve the resultant models. But the absence of future data on these variables could limit their use for studies which assess the effect of climate change on the current distribution models.

## Conclusion

GIS techniques along with climatological variables could help in developing models to assess the habitat suitability of specific insect pest species. In this study, we have successfully modeled the current and future global distribution of OWSF. With a spatial resolution of 5 km^2^ throughout the world, the models indicated current areas at risk and other regions with suitable habitat that could undergo new invasions by OWSF in the future. These patterns of models could help decision-makers and quarantine authorities to expedite control programs for such pests which have serious impact on human health and livestock industry. They also pave the way to furthers investigations on a local scale, especially for areas predicated to be highly suitable, with the incorporation of ecological criteria such as altitude and anthropogenic factors in addition to climatological factors for modeling this interesting veterinary pest. It is important that records of occurrence and georeferencing locations should be shared globally through a common-language database, as a paucity of records could impede successful modeling strategies^59^.

## Supplementary information

Supplementary information

Supplementary information2
