# Supplementary material for: Modeling current and future global distribution of Chrysomya bezziana under changing climate
Source: Sci Rep. 2020 Mar 18;10:4947. doi: 10.1038/s41598-020-61962-8 (PMC7080715; doi:10.1038/s41598-020-61962-8)
Supplement: Supplementary file 1 — Supplementary information [file 41598_2020_61962_MOESM1_ESM.pdf]

Modeling current and future global distribution of *Chrysomya bezziana* under changing climate

Eslam M. Hosni[1]*, Mohamed G. Nasser[1], Sara A. Al-Ashaal[1], Magda H. Rady[1], Mohamed A. Kenawy[1]

[1] *Department of Entomology, Faculty of Science, Ain Shams University, Abbassia, Cairo 11566, Egypt*

***Corresponding author at:** Department of Entomology, Faculty of Science, Ain Shams University, Abbassia, Cairo 11566, Egypt.

E-mail: iobek@sci.asu.edu.eg

Supplementary information S1

The current bioclimatic factors can be downloaded from the link below:

https://www.worldclim.org/current.

We used:

ESRI grids

bioclimatic variables

2.5 minutes “5 Km^2 spatial distance”.

Supplementary information S2

The future representative concentration pathways (RCPs) can be downloaded from the link below:

https://www.worldclim.org/CMIP5v1

We choose:

2.5 minutes “5 Km^2 spatial distance”.

https://www.worldclim.org/cmip5_2.5m

For time period 2050:

GCM	Code	RCP 2.6	RCP 8.5
MRI-CGCM3	MG	bi*	bi*

*To get the 19 bioclimatic factors.

For time period 2070:

GCM	Code	RCP 2.6	RCP 8.5
MRI-CGCM3	MG	bi*	bi*

*To get the 19 bioclimatic factors.

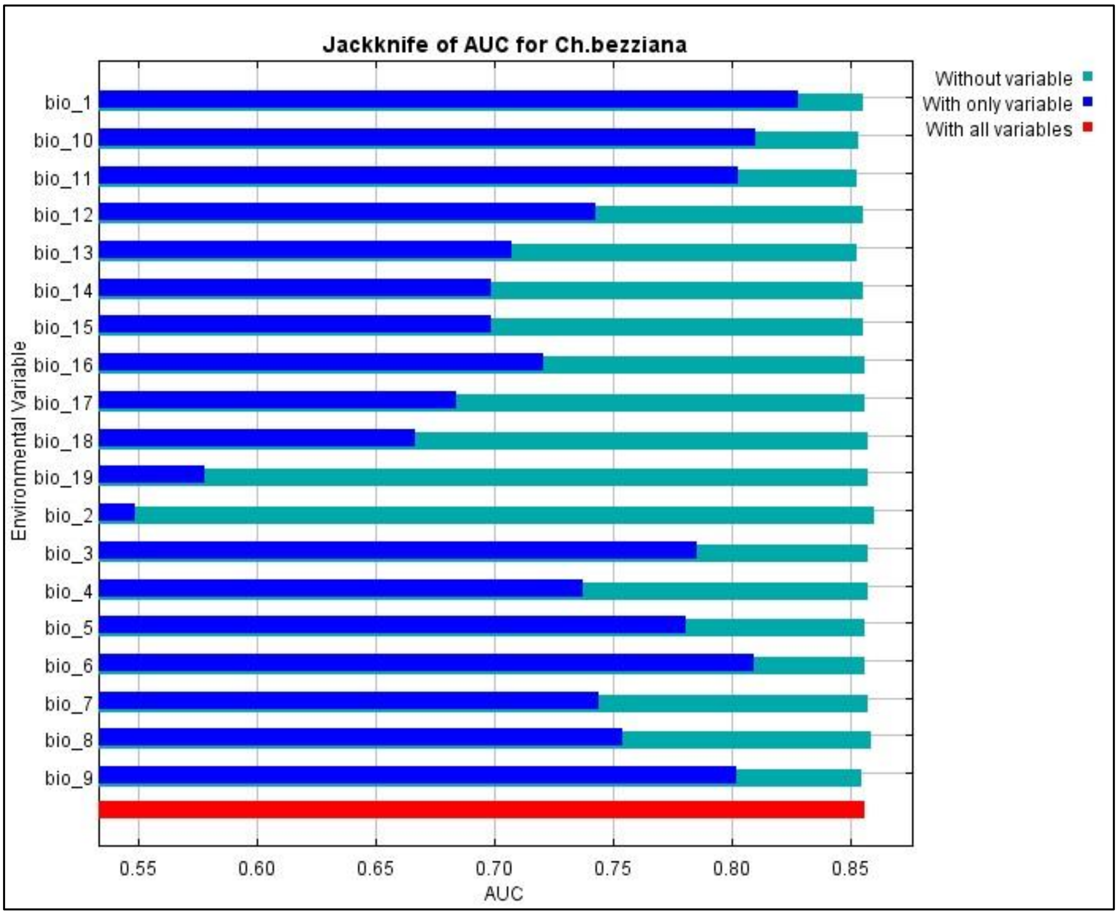

Supplementary Figure S3. Primary Jackknife test illustrating the most contributing bioclimatic variables.

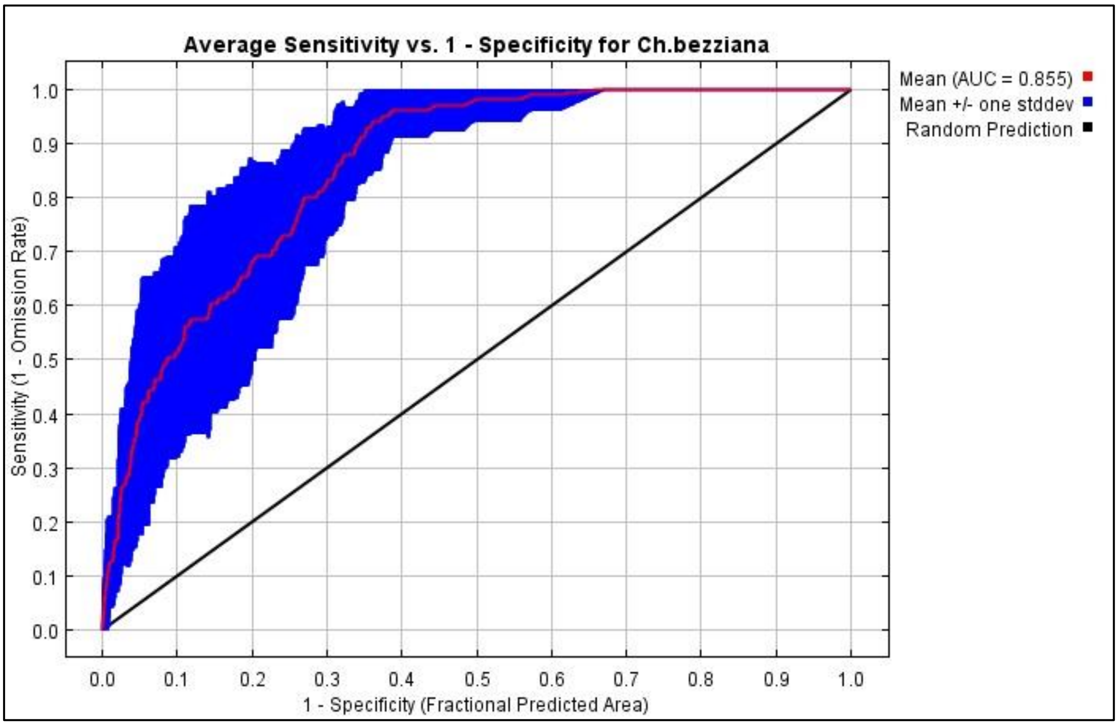

Supplementary Figure S4. Graphical result of AUC test.

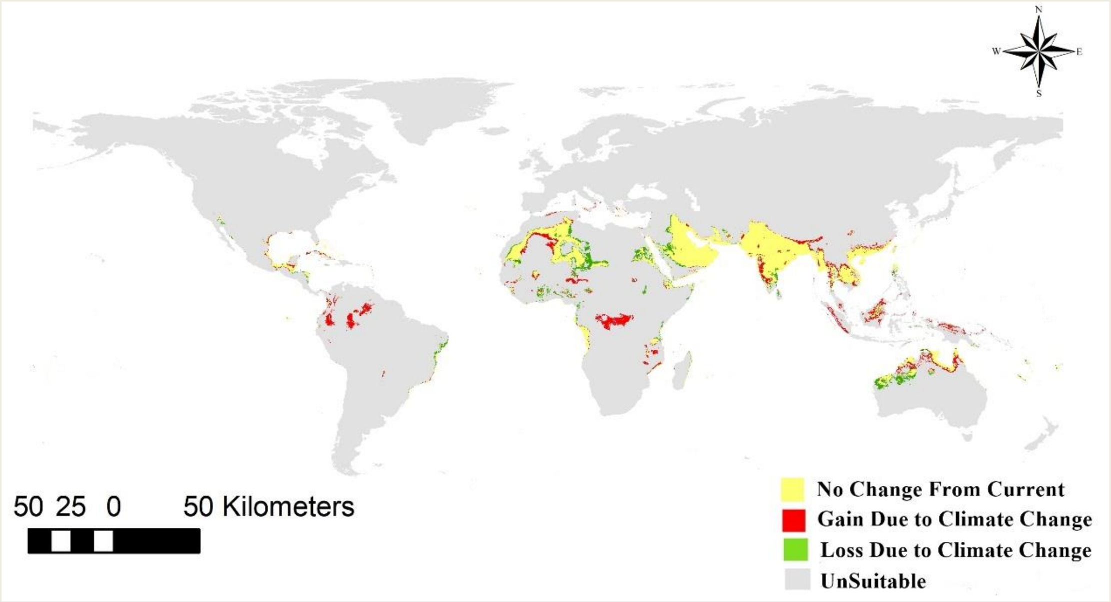

Supplementary Figure S5. Interactive thresholding map shows gain and loss habitat suitability differences between current and future distribution models of *C. bezziana* at RCP 2.6 in 2050.

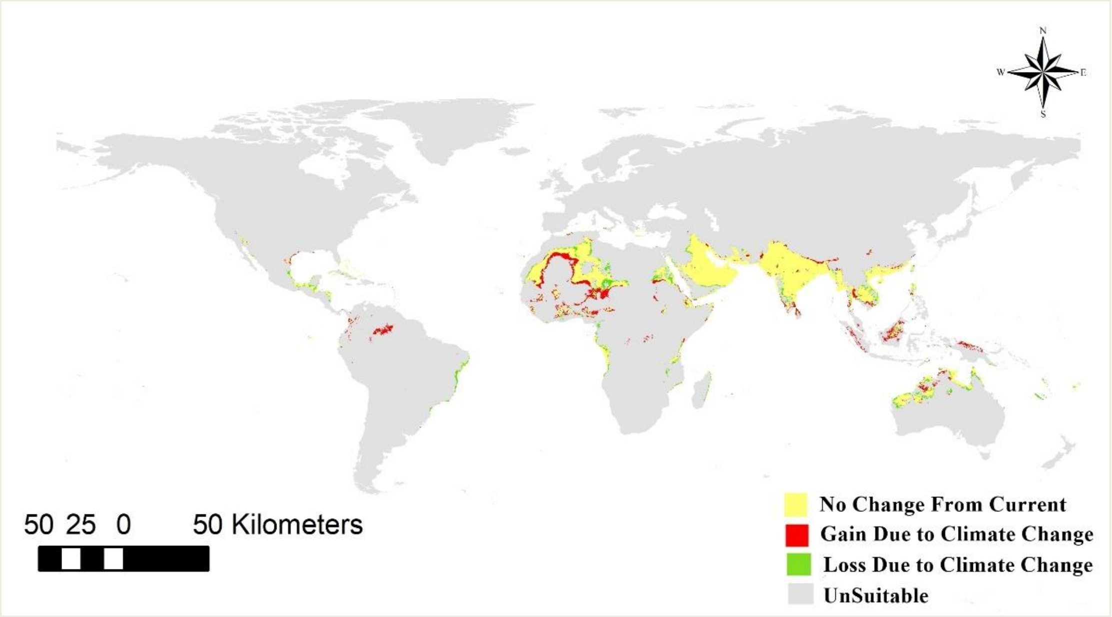

Supplementary Figure S6. Interactive thresholding map shows gain and loss habitat suitability differences between current and future distribution models of *C. bezziana* at RCP 8.5 in 2050.

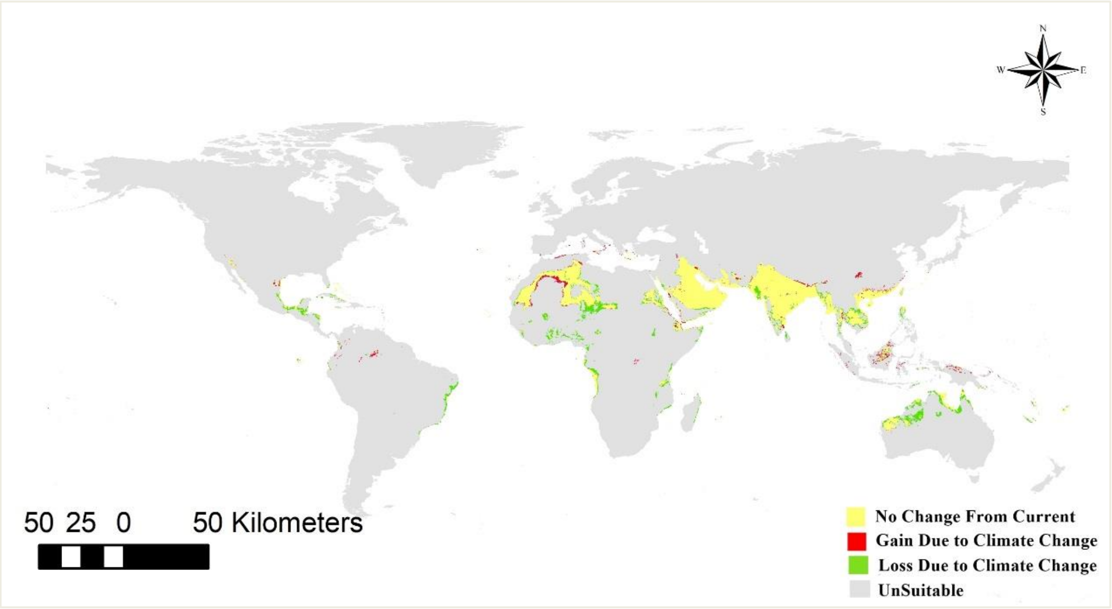

Supplementary Figure S7. Interactive thresholding map shows gain and loss habitat suitability differences between current and future distribution models of *C. bezziana* at RCP 2.6 in 2070.

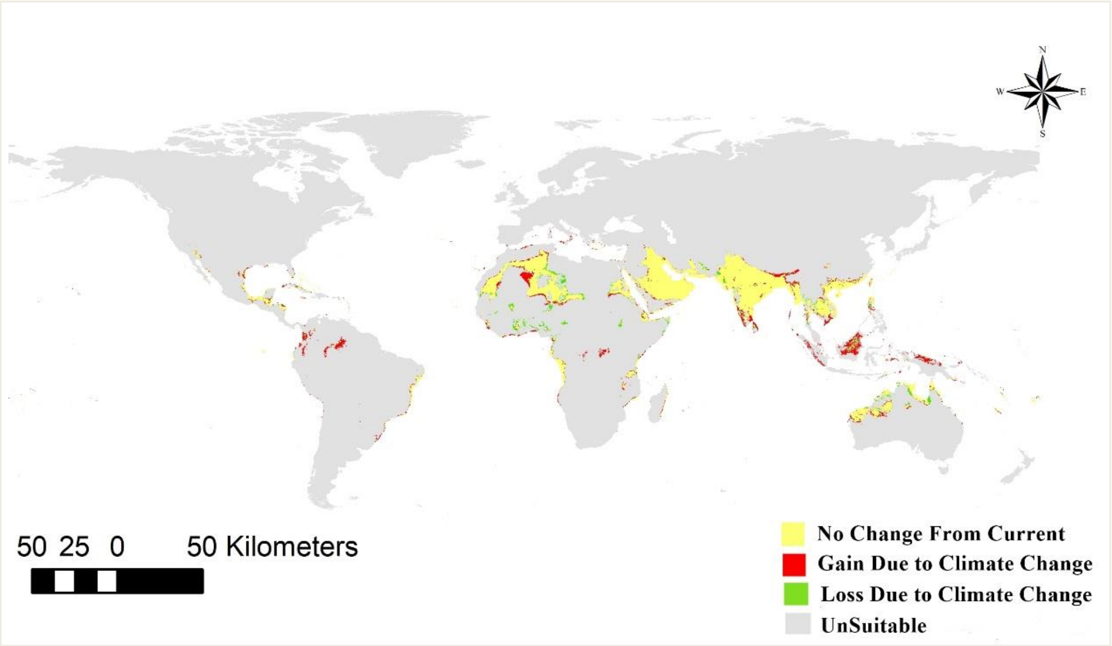

Supplementary Figure S8. Interactive thresholding map shows gain and loss habitat suitability differences between current and future distribution models of *C. bezziana* at RCP 8.5 in 2070.

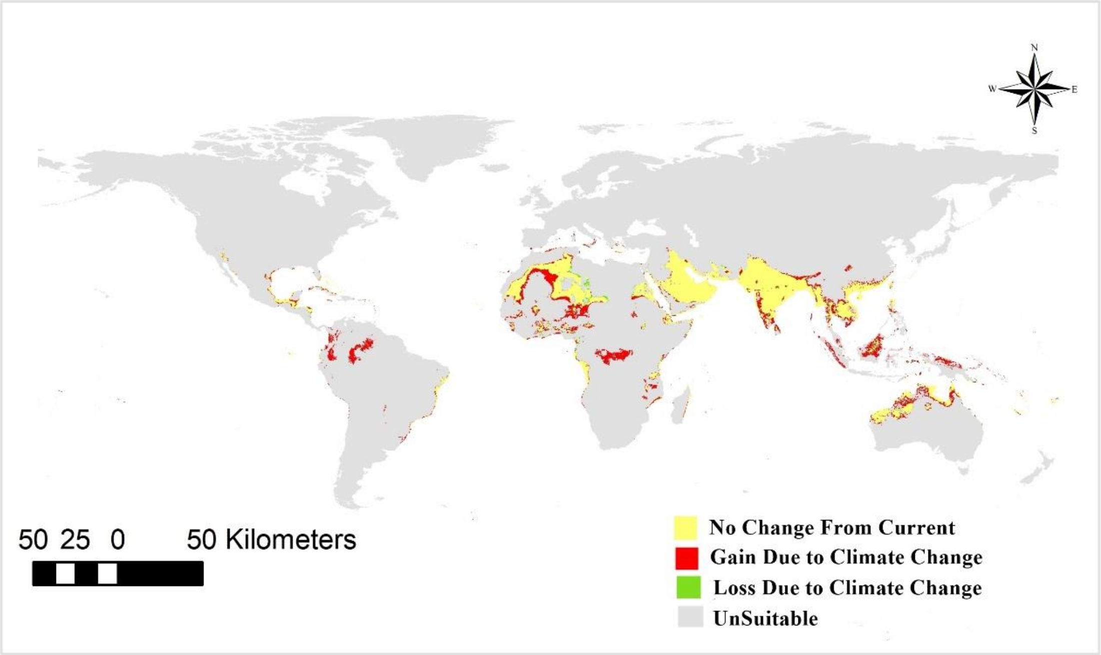

Supplementary Figure S9. Interactive thresholding map for overall gain and loss habitat suitability differences among current and future models.
